# A case of disseminated carcinomatosis of the bone marrow from gastric cancer developing rapidly after a gastrectomy

**DOI:** 10.1186/s40792-021-01135-5

**Published:** 2021-02-16

**Authors:** Shunsuke Sato, Yuji Ishibashi, Koichiro Kawasaki, Ryoto Yamazaki, Fumihiko Hatao, Yasuhiro Morita, Kazuhiro Imamura

**Affiliations:** 1 Department of Surgery, Tokyo Metropolitan Tama Medical Center, 2-8-29 Musashidai, Fuchu, Tokyo 183-8524 Japan; 2 Department of Surgery, Tokyo Metropolitan Matsuzawa Hospital, 2-1-1 Kamikitazawa, Setagaya City, Tokyo 156-0057 Japan

**Keywords:** Bone marrow metastasis, Disseminated carcinomatosis of the bone marrow, Gastrectomy, Gastric cancer

## Abstract

**Background:**

Disseminated carcinomatosis of the bone marrow (DCBM) is often associated with disseminated intravascular coagulation (DIC) and a poor prognosis. Moreover, the timing of the diagnosis varies. We presented herein the first report of a case of DCBM from gastric cancer that developed rapidly after a gastrectomy.

**Case presentation:**

A 42-year-old male patient was referred to us for gastric cancer. Preoperative laboratory tests were normal. Abdominal computed tomography (CT) revealed no obvious bone metastasis, and he underwent a laparoscopic distal gastrectomy. On postoperative day (POD) 1, laboratory data indicated severe thrombocytopenia. Postoperative bleeding requiring reoperation was found. Afterwards, he complained of lower back pain. His ALP and LDH gradually became elevated. On POD 8, DIC was diagnosed. CT and bone scintigraphy showed multiple, widespread bone metastases. Based on these findings, DCBM from gastric cancer was diagnosed. Systemic chemotherapy was started on POD 12. The DIC subsided during the first course, and he was discharged on POD 21. The patient died of tumor progression 7 months later.

**Conclusion:**

When thrombocytopenia is observed immediately after a gastrectomy for gastric cancer, the possibility of DCBM should be considered.

## Introduction

Bone metastasis diffusely invading the bone marrow with attendant hematological disorders is called disseminated carcinomatosis of the bone marrow (DCBM). DCBM is often associated with disseminated intravascular coagulation (DIC) and a poor prognosis [1]. The timing of diagnosis of the bone and bone marrow metastases varies, and the median interval from the diagnosis of gastric cancer to the detection of a bone marrow metastasis was previously reported as 161 days [2]. Herein, we reported the first case of DCBM associated with gastric cancer that developed rapidly after a gastrectomy.

## Case report

**Fig. 1:**
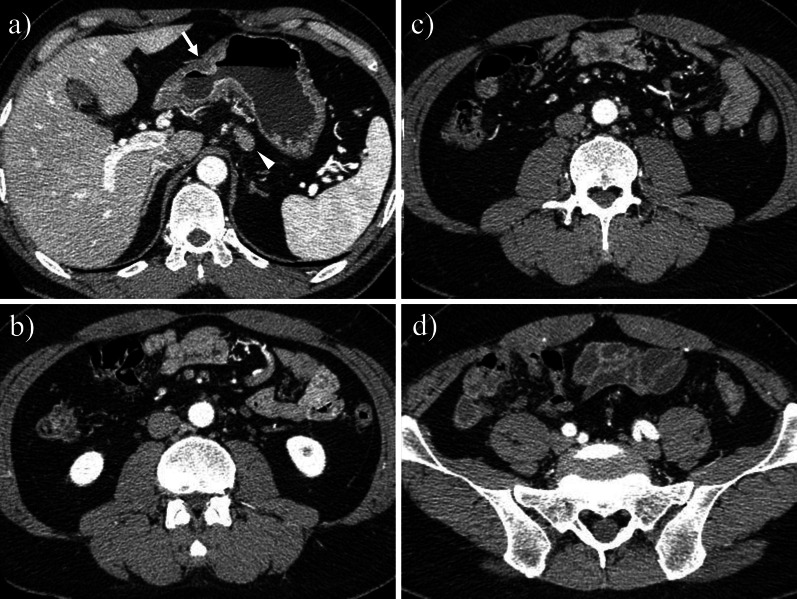
Preoperative CT. **a** CT revealed wall thickening with enhancement of the gastric angle (arrow) and regional lymph node swelling (arrowhead). **b**–**d** No obvious bone metastasis was observed

**Fig. 2:**
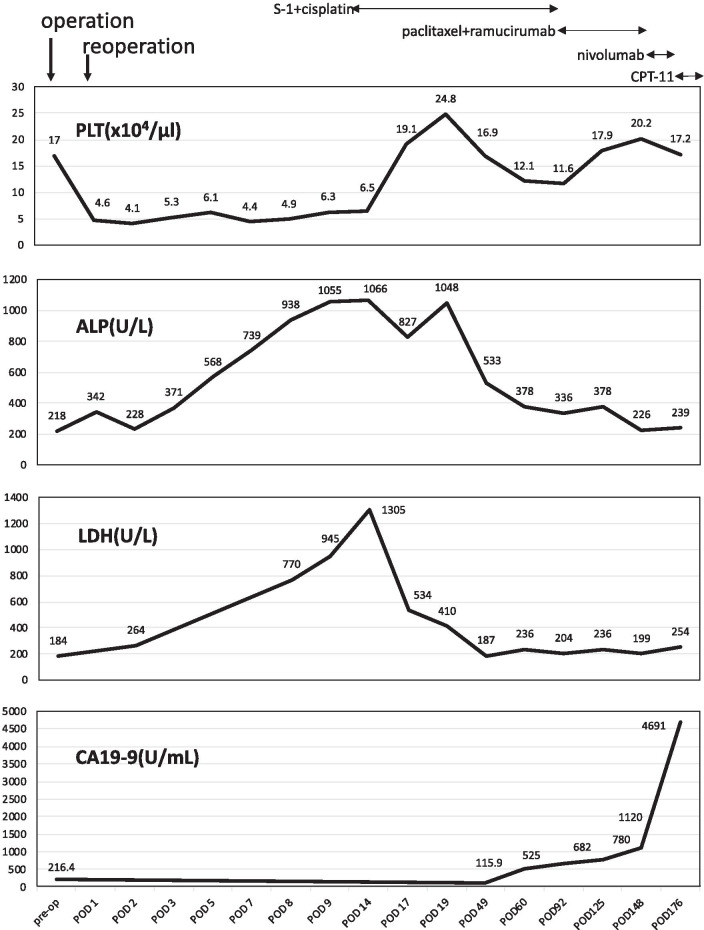
Clinical course of the patient including the surgery, chemotherapy, and clinical data

**Fig. 3:**
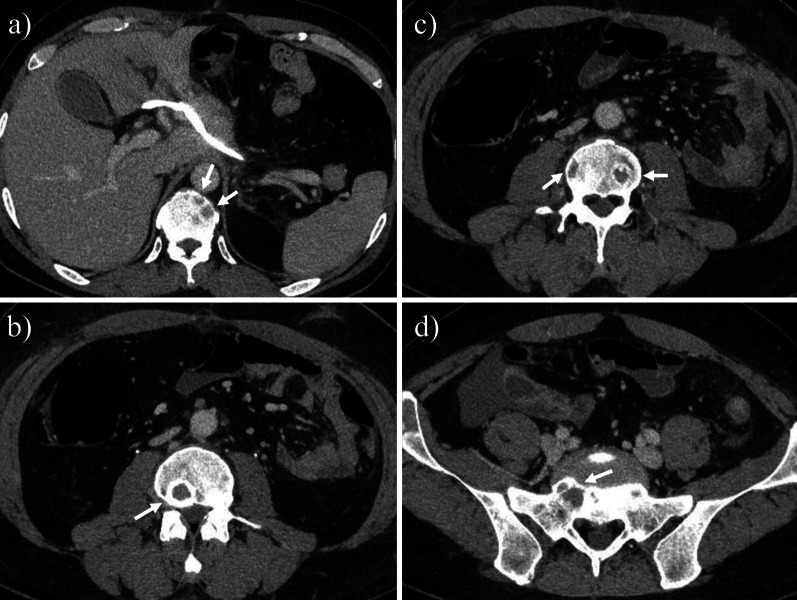
Postoperative CT. Multiple, widespread, low-density masses were seen in the vertebrae (arrow)

**Fig. 4:**
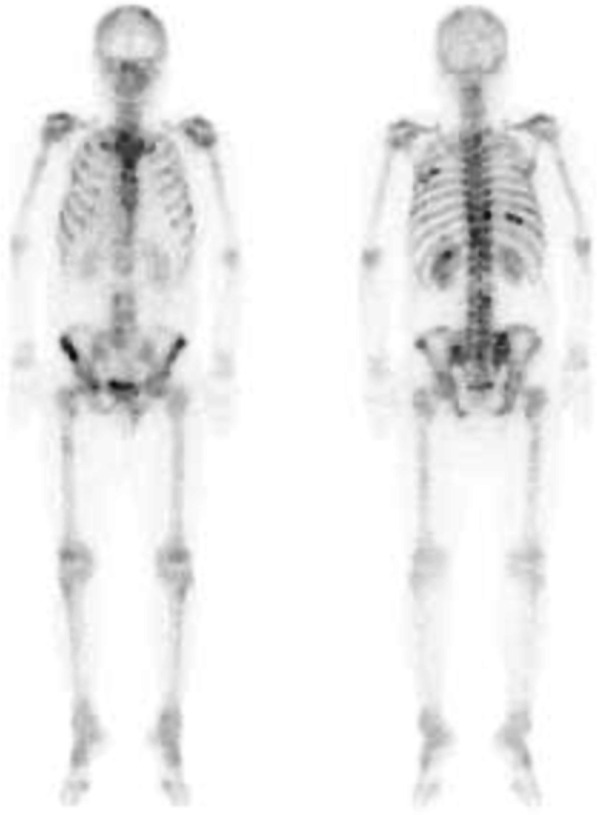
Bone scintigraphy revealed multiple hot spots throughout most of the spine, pelvis, and ribs

A 42-year-old male patient was referred to our department for gastric cancer. He was asymptomatic on admission. A laboratory test on preoperative day 13 showed elevated carbohydrate antigen (CA) 19-9. Other laboratory values, including platelet count (PLT), alkaline phosphatase (ALP), and lactate dehydrogenase (LDH), were normal (Table 1). Upper gastrointestinal endoscopy showed a type 3 lesion on the lesser curvature of the gastric angle. Pathological analysis of a biopsy specimen demonstrated a poorly differentiated adenocarcinoma. Abdominal computed tomography (CT) performed at the previous hospital on preoperative day 20 revealed wall thickening with enhancement of the gastric angle and some regional lymph node swelling. Bone metastasis was not diagnosed (Fig. 1). Based on these findings, clinical stage III gastric cancer (cT3N + M0) was diagnosed based on the Japanese Gastric Cancer Association Classification, 14th Edition [3]. The patient underwent a laparoscopic distal gastrectomy and D2 lymph node dissection. The total duration of the surgery was 305 min, and the blood loss was 120 ml. No bleeding disorder was found during surgery and no intraoperative blood transfusion was required. On postoperative day (POD) 1, the drainage showed evidence of hemorrhage. Laboratory data indicated severe thrombocytopenia (4.6 × 10^4^/µl). Continuous bleeding requiring reoperation was found. Exploratory laparoscopy was performed. The intraoperative findings demonstrated hematomas around the greater omentum, and the points of bleeding were some stumps of the omentum. Lavage and hemostasis were performed under laparoscopy. After reoperation, the bleeding did not recur, but the patient complained of lower back pain. His PLT remained low while his ALP and LDH gradually became elevated (Fig. 2). Findings on POD 8 showed PLT 6.3 × 10^4^/µl, ALP 1055 U/l (ALP-1: 23%, ALP2 + 3: 77%), LDH 945 U/l, d-dimer 150 µg/ml, and FDP 296 µg/ml (Table 1). Based on these findings, DIC was diagnosed. CT showed multiple, widespread, low-density masses in the vertebrae which were not detected on preoperative CT, and bone scintigraphy revealed multiple hot spots throughout most of the spine, pelvis, and ribs (Figs. 3, 4). Although a bone biopsy was not able to be performed because of the patient’s poor condition, DCBM from gastric cancer was clinically diagnosed. The histopathological examination revealed the following: L, Less, Type3, 51 × 27 mm, por2 > tub2, pT3(SS), INFc, Ly1c, V1a, pPM0, pDM0, pN3b (42/53). Human epidermal growth factor receptor 2 was negative. The final diagnosis was T3N3bM1 Stage IV.

**Table 1:** Preoperative and postoperative laboratory data

	Preoperative	POD 1	POD 8	POD 19
WBC (/µl)	7500	10,000	14,800	4400
RBC (× 10^4^/µl)	532	411	337	306
Hb (g/dl)	15	11.7	9.7	9.0
Ht (%)	43.9	34.3	28.9	26.7
PLT (× 10^4^/µl)	17	4.6	6.3	24.8
PT (%)	84.1	–	38.6	74.7
APTT (s)	27.3	–	33.9	23.4
Fib (mg/dl)	289	–	171	
FDP (µg/ml)	–	–	296	85.4
d-Dimer (µg/ml)	–	–	150	34.2
TP (g/dl)	7.2	–	6.3	5.8
Alb (g/dl)	4.1	3.1	3.2	3.4
T-Bil (mg/dl)	0.8	1	1.2	0.6
AST (U/l)	16	232	44	19
ALT (U/l)	13	288	60	20
LDH (U/l)	184	-	945	410
ALP (U/l)	218	342	1055	1048
BUN (mg/dl)	11.1	20.5	11.5	14.8
Cr (mg/dl)	0.7	1.13	0.62	0.63
CRP (mg/dL)	2.04	7.04	7.51	0.33
CEA (ng/ml)	2.9	–	–	–
CA19-9 (U/ml)	216.4	–	–	–

*POD* postoperative day

Systemic chemotherapy with S-1 + cisplatin (SP) was started on POD 12. The DIC subsided during the first course, and the patient was discharged on POD 21 (Table 1, Fig. 2). Radiation 30 Gy in ten fractions for the bone metastases was started on POD 24. After two cycles of SP, the CA19-9 levels rose again. Thereafter, he received two courses of paclitaxel and ramucirumab as the second-line treatment, two courses of nivolumab as the third line treatment, and one course of CPT-11 as the fourth line treatment. However, these regimens did not achieve a curative effect. The patient died seven months postoperatively.

## Discussion

Gastric cancer most commonly spreads to the peritoneum, liver or lungs. The incidence of bone metastasis is 2–3% [4, 5]. A bone marrow metastasis is rare, with an incidence of less than 1% [6]. In DCBM, cancer cells diffusely infiltrate the bone marrow, then proliferate explosively, causing bone destruction and hematological complications [1]. Elevated expression of receptor activator of nuclear factor kappa-B ligand in the gastric tissues plays an important role in DCBM pathogenesis [1]. The majority of DCBM patients are of younger age with histologically poorly differentiated adenocarcinoma or signet ring cell carcinoma, elevated ALP and LDH, thrombocytopenia, anemia, and extensive bone metastases [2]. The patient in the present case had gastric cancer with several, typical features of DCBM, such as poorly differentiated adenocarcinoma, thrombocytopenia, elevated serum ALP and LDH, widespread bone metastasis, and DIC, based on which DCBM from gastric cancer was clinically diagnosed.

The prognosis of DCBM is extremely poor. Park et al. reported that patients with a bone marrow metastasis had significantly poor survival compared to those without a bone marrow metastasis [5]. Kim et al. reported that the median survival time of patients with both bone and bone marrow metastases was 4.0 months while that of patients with only a bone metastasis was 8.7 months [2]. The present patient died only seven months after surgery despite intensive chemotherapy.

The timing of the diagnosis of bone and bone marrow metastases varies. Turkoz et al. reported that the median duration from the diagnosis of gastric cancer to the detection of a bone metastasis was 14.2 months in resected gastric cancer patients and 8.2 months (range 2.5–121.1 months) in inoperable gastric cancer patients [4]. On the other hand, Kim et al. reported that the median interval from the diagnosis of gastric cancer to the detection of a bone marrow metastasis was 161 days (range 0–2860 days) [2]. The present patient was asymptomatic, his serum PLT, ALP, and LDH levels were normal, bone metastasis was not diagnosed by preoperative CT, and no bleeding disorder was found during laparoscopic gastrectomy. Immediately after the gastrectomy, thrombocytopenia suddenly developed; a hemorrhage requiring reoperation was found, and the ALP and LDH became elevated. CT and bone scintigraphy showed multiple bone metastases, leading to the diagnosis of DCBM which developed rapidly after the gastrectomy. No similar cases have been reported previously. Although in the present case, the accurate cause and mechanism by which the bone metastasis and DCBM progressed rapidly almost immediately after surgery were unclear, it is possible that a subclinical bone micrometastasis escaped detection on CT before his first operation and operative invasiveness and the stress induced by the gastrectomy may have contributed to the rapid and diffuse progression of bone metastasis, causing the cancer cells to infiltrate the bone marrow where they proliferated explosively and developed into DCBM. Furthermore, it is possible that reoperation for the postoperative bleeding increased the surgery-induced stress in this patient. The body’s response to surgery-induced stress has direct effects on tumor cells or can alter the tumor microenvironment, increasing the risk of recurrence [7]. Also, surgery can lead to the activation of early and key components of the innate and adaptative immune systems, and platelet activation, neutrophil extracellular traps, and immunosuppression are significant factors in promoting tumor growth and metastasis [8].

## Conclusion

We reported the first case of DCBM associated with gastric cancer that developed rapidly after a gastrectomy. The possibility of DCBM should be considered in cases of thrombocytopenia occurring immediately after a gastrectomy for advanced gastric cancer.

## Data Availability

Data in the study are not available to the public due to concerns about patient privacy but may be obtained from the corresponding author on reasonable request.

## Abbreviations

DCBM: Disseminated carcinomatosis of the bone marrow
DIC: Disseminated intravascular coagulation
CA 19-9: Cardioantigen 19-9
PLT: Platelet count
ALP: Alkaline phosphatase
LDH: Lactate dehydrogenase
CT: Computed tomography
POD: Postoperative day
SP: S-1 + cisplatin
